# Effects of Preserving the Pulmonary Vagus Nerve Branches on Cough After Pneumonectomy During Video-Assisted Thoracic Surgery

**DOI:** 10.3389/fonc.2022.837413

**Published:** 2022-03-09

**Authors:** Shaorui Gu, Wenli Wang, Xishi Wang, Kaiqin Wu, Xin Zhang, Shiliang Xie, Yongxin Zhou

**Affiliations:** Department of Thoracic Surgery, Shanghai Tongji Hospital, School of Medicine, Tongji University, Shanghai, China

**Keywords:** CAP, preservation of pulmonary vagus nerve branches, VATS, early-stage non-small cell lung cancer (early-stage NSCLC), postoperative complications

## Abstract

**Background:**

Cough is one of the most common complications of early-stage non-small cell lung cancer (NSCLC) after video-assisted thoracoscopic surgery (VATS). The vagus nerve plays an important role in pulmonary inflammation and the cough reflex. In this study, we attempted to reduce the incidence of postoperative chronic cough and other complications by preserving the pulmonary vagus nerve branches.

**Patients and Methods:**

This study was a randomized controlled double-blinded trial of subjects and observers. A total of 158 NSCLC patients were enrolled. We randomly assigned 79 patients to Group A (pulmonary branch of vagus nerve preservation group) and 79 cases to Group B (conventional surgical treatment group). In the final analysis, 72 patients from Group A and 69 patients from Group B were included. The main outcome measure of the study was the occurrence of CAP or other postoperative complications within five weeks. This trial was registered with ClinicalTrials.gov (number NCT03921828).

**Results:**

There was no significant difference in preoperative general clinical data between the two groups. No death during the perioperative period occurred in either of the two groups. There was no significant difference between the two groups in operation time, intraoperative bleeding, number of lymph nodes sent for examination, number of cases transferred to ICU after operation, postoperative catheterization time, or postoperative hospital stay (P>0.05). There was no significant difference in other pulmonary and cardiovascular complications between the two groups, including pulmonary infection (2.78% vs. 8.70%, P = 0.129), atelectasis (1.39% vs. 0%, P = 0.326), pleural effusion (2.78% vs. 1.45%, P = 0.585), persistent pulmonary leakage (2.78% vs. 2.90%, P = 0.965), arrhythmia (2.78% vs. 1.45%, P = 0.585), and heart failure (0% vs. 1.45%, P = 0.305). The incidence of CAP in Group A was significantly lower than that in Group B (13.89% vs. 30.43%, P = 0.018). The LCQ-MC scores in Group A were significantly higher than those in Group B at two and five weeks after operation (P<0.05). Univariate and multivariate analysis showed that the risk factors for postoperative CAP were surgical side (right lung), surgical lung lobe (upper lobe), preservation of pulmonary branch of the vagus nerve during operation, and duration of anesthesia.

**Conclusions:**

Preserving the pulmonary vagus nerve branches during VATS in patients with stage IA1-2 NSCLC can reduce the incidence of postoperative CAP.

## Introduction

In recent years, video assisted thoracic surgery (VATS) has been widely used, meaning that the majority of early lung cancer patients receive improved treatment (1). The most common type of complication is cough after pneumonectomy (CAP), which accounts for 25% ~ 50% of all the surgical patients (2). CAP is defined as a dry cough lasting no less than two weeks following pneumonectomy with no obvious abnormality present in a chest x-ray (3). The effects of factors such as postnasal drip syndrome, bronchial asthma, and oral angiotensin converting enzyme inhibitor (ACEI) drugs are excluded (4).

Sensory neurons of the vagus nerve are the main source of nerve fibers innervating the lung and airway, which are very important for normal breathing (5). Previous studies have shown that the vagus nerve serves important roles in regulating lung function, such as in the cough reflex, mucus secretion, and bronchial diameter (6). In addition, the vagus nerve is involved in the regulation of inflammation (7). Many other factors causing pulmonary complication, such as choking, aspiration, phlegm, and blood stasis, also have causal effects on the disconnection and loss of the vagus nerve during operation (8).

For a long period of time, research on the correlation between the pulmonary vagus nerve branches and pulmonary complication has been stagnant due to the lack of accurate anatomy of the pulmonary vagus nerve branches (9). Wejis took the lead in addressing this issue by exploring the anatomy of six cadavers, drawing an accurate anatomical map of the pulmonary vagus nerve branches on this basis, and successfully dissecting ten cadavers under thoracoscopy, ultimately suggesting the feasibility of preserving pulmonary vagus nerve branches nerve during operation (10).

In order to explore the relationship between the pulmonary vagus nerve and postoperative complications of lung cancer, we reserved the pulmonary vagus nerve branches during thoracoscopic surgery for early lung cancer to clarify its impact on CAP and other complications.

## Materials and Methods

### Study Design and Population

We performed a randomized and double-blinded controlled trial of patients and observers. All patients underwent thoracoscopic pneumonectomy in the Department of Cardiothoracic Surgery of Shanghai Tongji Hospital. This experimental study was approved by the ethics committee of Shanghai Tongji Hospital. Patients were screened using chest CT scans. Preoperative clinical stages were T1a-bN0M0 or Ia1-2. The postoperative pathological diagnosis was non-small cell lung cancer. If there was massive bleeding during the operation, or if the postoperative pathology indicated benign tumor or inflammatory focus, the patient was removed from the group. All subjects were aware of the purpose of the study, were able to comply with the requirements of the study, and provided signed informed consent prior to operation.

### Randomization and Masking

The study nurse was responsible for registering eligible patients. According to random number assignment, eligible patients were divided in a 1:1 ratio into the following two groups: the pulmonary branch of the vagus nerve preservation group (Group A) and the conventional surgical treatment group (Group B). Random numbers were generated using excel tables. The main investigator signed the informed consent form with the patient or their family one day before the operation and informed the surgeon about the grouping of the patient. The experimental researchers randomly selected one of the two surgeons for surgery. Postoperative patient management was completed by two other physicians who did not know the grouping of patients. There was no significant difference in postoperative medication used between the two groups. Surgeons were allowed to visit patients after surgery, but were not able to give any orders. The time for removing the chest catheter was when there was no obvious gas emission after the patient coughed and the drainage flow was less than 200 ml within 24h (11). Postoperative follow-up was completed by the observer. Bedside inquiry was mainly used during the first week after operation, whereas telephone follow-up or outpatient follow-up were used to complete the questionnaire two to five weeks after operation.

### Surgical Procedure and Postoperative Therapy

The patient was placed in the healthy lateral position with double lumen endotracheal intubation and one lung ventilation. The seventh or eighth intercostal space of the axillary midline was selected as the observation hole, and the fourth intercostal space of the axillary front was selected as the main operation hole with the aid of endoscopy to make a 2-4 cm small incision. Different surgical methods were selected according to the location of the tumor and intraoperative freezing, including wedge resection, segmental resection, and lobectomy (12).

Lymph nodes were grouped by the American Joint Committee on Cancer (AJCC) criteria and were sampled according to the NCCN guidelines for non-small cell lung cancer (2018) (13). We routinely sampled lymph nodes in groups 4, 5, 6, 7, 8, and 9 during left lung surgery. Meanwhile, lymph nodes in groups 2, 4, 7, 8, and 9 were routinely sampled during right lung surgery (14).

In Group B, the location of the pulmonary vagus nerve branch was determined according to anatomy during operation. Incision of pleura and lymph node area. The routine lymph node sampling method was used for sampling, and the pulmonary branches of the vagus nerve were not distinguished.

In Group A, pleurotomy was used to identify neural structures. Sampling was performed around the lymph nodes on the operation side to avoid injury to the pulmonary branch of the vagus nerve. Additionally, we only sampled the excised lymph nodes to preserve the vagus nerve.

After the operation, a 28-F drainage tube was placed in the patient’s thoracic cavity.

### Follow-Up Evaluation

We observed the incidence of pulmonary complications in the two groups within five weeks of operation, where complications included CAP, pulmonary infection, atelectasis, pleural effusion, postoperative respiratory failure, ARDS, and the need for endotracheal intubation. Other perioperative evaluation indexes included postoperative drainage time, postoperative mortality, incidence of postoperative cardiovascular complications, endotracheal intubation rate, ICU admission rate, ICU admission time, hospital stay, and hospitalization expenses.

We used the Mandarin Chinese version of the Leicester Cough Questionnaire (LCQ-MC) and visual simulation scale (VAS) score evaluation to determine the occurrence of CAP. The LCQ-MC included psychological, physiological, and social aspects, with a total score ranging between 3-21 (15). It is widely used in the evaluation of chronic cough. The focus of the questionnaire is to objectively and comprehensively evaluate the severity of postoperative chronic cough. The main scoring method of the VAS is to ask patients to select a number between 0-100 mm to represent the severity of their 24-hour cough (16). Patients with a VAS score of 60 mm or greater were classified into the cough group.

### Statistical Analysis

Data was input into an Excel table and SPSS Version 21.0 software was used for statistical analysis. A significance level of α= 0.05 was used for comparison between groups. Measurement data was described as mean ± SD, where the number of cases and percentage (%) of total were also expressed. The measurement data were compared between groups and analyzed using a two sample t-test. Chi-square or Fisher’s exact tests were used for comparison between groups involving count data. Multivariate correlation analysis was analyzed by binary logistic regression modeling. The results were expressed as corrected odds ratios (ORs) and 95% confidence intervals (CIs). P< 0.05 was considered statistically significant.

## Results

### General Clinical Data and Perioperative Information of Patients

**Figure 1:**
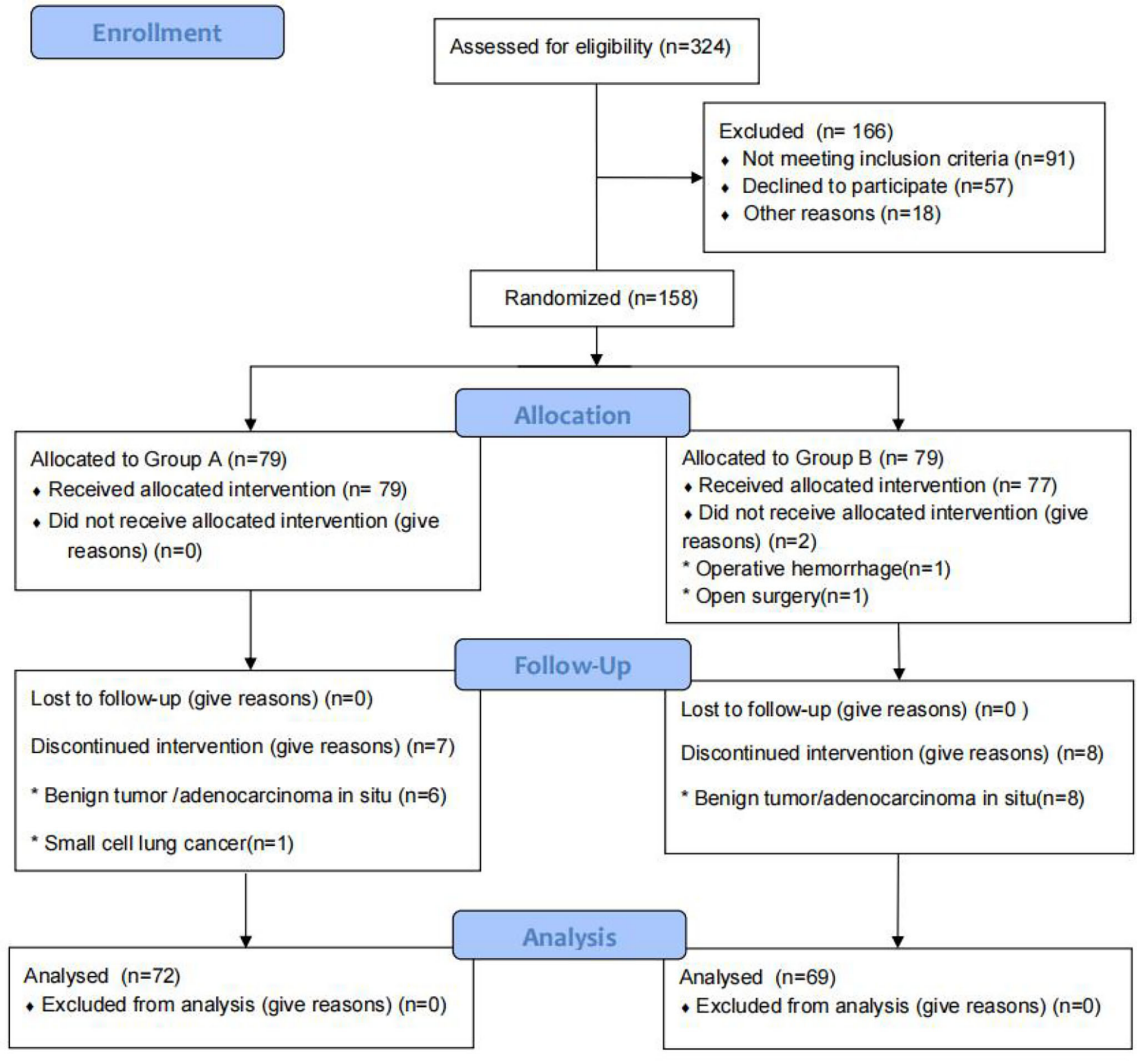
Patient flowchart.

From January 2019 to May 2021, we screened 324 patients with pulmonary nodules less than 2 cm on chest CT scans. After excluding 91 patients who did not meet the study criteria, we identified 233 patients for enrollment. Among this group, 57 patients refused to participate in the experiment and 18 patients did not participate for other reasons. Of the remaining 158 patients, 79 were assigned to the pulmonary branch of the vagus nerve preservation group (Group A) and 79 to the conventional surgical treatment group (Group B). In Group A, a total of seven patients (six with benign tumors or adenocarcinoma *in situ* and one with small cell lung cancer) were excluded from the final analysis. In Group B, a total of 10 patients (eight with benign tumors or adenocarcinoma in situ, one case of intraoperative conversion to open surgery, and one with massive intraoperative bleeding) were excluded from the final analysis. There were no patients lost to follow-up in this study. There was no significant difference in gender, age, tumor location, pathology, or history of past illness (smoking history, hypertension, diabetes, or coronary heart disease) between the two groups (P > 0.05). Baseline characteristics are summarized in **Table 1** and **Figure 1**.

**Table 1:** Comparison of preoperative general data between the two groups.

General information		Group A	Group B	x^2^	P value
n		72	69		
Gender				0.013	0.908
	Male	40 (55.6)	39 (56.5)		
	Female	32 (44.4)	30 (43.5)		
Age (years)				0.354	0.552
	<60	35 (48.6)	37 (53.6)		
	≥60	37 (51.4)	32 (46.4)		
History of past illness					
	Hypertension	23 (31.9)	20 (29.0)	0.146	0.703
	Coronary heart disease	5 (6.9)	8 (11.6)	0.910	0.340
	Diabetes	7 (9.7)	11 (15.9)	1.224	0.269
	Smoking history	23 (31.9)	20 (29.0)	0.146	0.703
Operated side				3.092	0.079
	Left lung	29 (40.3)	38 (55.1)		
	Right lung	43 (59.7)	31 (44.9)		
Tumor site				0.157	0.692
	Upper lung	41 (56.9)	37 (53.6)		
	Middle and lower lung	31 (43.1)	32 (46.4)		
Pathology				2.299	0.585
	Adenocarcinoma	70 (97.2)	68 (98.6)		
	Squamous cell carcinoma	2 (2.8)	1 (1.4)		
Pulmonary resection type				0.199	0.655
	Lobectomy	38 (52.8)	39 (56.5)		
	Subpulmonary lobectomy	34 (47.2)	30 (43.5)		

There were no perioperative deaths in either of the two groups. There was no significant difference in perioperative indexes between the two groups, where these indexes included operation time, intraoperative bleeding, number of lymph nodes sent for examination, number of patients transferred to ICU after operation, time of catheterization after operation, and postoperative hospital stay (P > 0.05). In terms of operation time, Group A took longer than Group B (115.069 ± 36.177 vs. 109.493 ± 34.215), but there was no significant difference (P > 0.05). These results are shown in **Table 2**.

**Table 2:** Comparison of perioperative conditions between the two groups.

Perioperative indicators	Group A	Group B	x^2^/t	P value
n	72	69		
Duration of anesthesia (min)	164.236 ± 34.820	163.986 ± 35.111	0.043	0.966
Operation time (min)	115.069 ± 36.177	109.493 ± 34.215	0.940	0.349
Catheterization after operation (day)	3.667 ± 1.233	3.797 ± 1.313	-0.608	0.544
The number of lymph nodes	6.222 ± 1.456	6.000 ± 1.706	0.833	0.406
Intraoperative bleeding (ml)	83.056 ± 34.176	89.130 ± 34.884	-1.044	0.298
Patients transferred to ICU	3 (4.2)	2 (2.9)	0.000	1.000
Postoperative hospital stay	5.375 ± 1.358	5.841 ± 1.763	-1.762	0.080

### Postoperative Cardiopulmonary Complications

The incidence of CAP in Group A was significantly lower than that in Group B (13.89% vs. 30.43%, P = 0.018). There was no significant difference in other pulmonary and cardiovascular complications between the two groups, including pulmonary infection (2.78% vs. 8.70%, P = 0.129), atelectasis (1.39% vs. 0%, P = 0.326), pleural effusion (2.78% vs. 1.45%, P = 0.585), persistent pulmonary leakage (2.78% vs. 2.90%, P = 0.965), arrhythmia (2.78% vs. 1.45%, P = 0.585), and heart failure (0% vs. 1.45%, P = 0.305). These results are shown in **Table 3**.

**Table 3:** Comparison of postoperative complications between the two groups.

General information	Group A	Group B	x^2^/Fisher	P value
n	72	69		
CAP	10 (13.89)	21 (30.43)	5.624	0.018^*^
Pulmonary infection	2 (2.78)	6 (8.70)	2.306	0.129
Atelectasis	1 (1.39)	0	0.965	0.326
Pleural effusion	2 (2.78)	1 (1.45)	0.299	0.585
Persistent pulmonary leak	2 (2.78)	2 (2.90)	0.002	0.966
Arrhythmia	2 (2.78)	1 (1.45)	0.299	0.585
Heart failure	0	1 (1.45)	1.051	0.305

^*^Statistically significant (p < 0.05).

### LCQ-MC Score Before and After Operation

There was no significant difference in LCQ-MC score between Group A and Group B before operation(P > 0.05). There were significant differences in the scores of physiology, psychology, and total points between the two groups two and five weeks after operation (P < 0.05), but there was no significant difference in the scores of sociology between the two groups (p > 0.05).The LCQ-MC score in Group A five weeks after operation was significantly higher than that at two weeks after operation except sociology. The situation of Group B was similar to that of Group A. These results are shown in **Table 4**.

**Table 4:** Comparison of the mean LCQ-MC score between the two groups before and after surgery.

Variables	LCQ-MC	Group A (n=72)	Group B (n=69)	t	P-value
Preoperative	Sociology	6.396 ± 0.278	6.377 ± 0.375	0.341	0.734
	Physiology	6.372 ± 0.205	6.270 ± 0.380	1.449	0.150
	Psychology	6.257 ± 0.305	6.302 ± 0.297	-0.878	0.382
	Total score	19.024 ± 0.494	18.945 ± 0.753	0.460	0.647
2 weeks after operation	Sociology	5.604 ± 0.436	5.562 ± 0.543	0.512	0.609
	Physiology	5.607 ± 0.403	5.453 ± 0.469	2.101	0.037*
	Psychology	5.689 ± 0.393	5.426 ± 0.458	3.656	0.000*
	Total score	16.900 ± 0.982	16.440 ± 1.210	2.481	0.014*
5 weeks after operation	Sociology	5.802 ± 0.398	5.783 ± 0.477	0.264	0.792
	Physiology	5.815 ± 0.405	5.642 ± 0.426	2.481	0.014*
	Psychology	5.849 ± 0.346	5.691 ± 0.406	2.497	0.014*
	Total score	17.467 ± 0.939	17.115 ± 1.044	2.104	0.037*

^*^Statistically significant (p < 0.05).

### Univariate Analysis of CAP

Univariate analysis of 31 cases in the cough group and 110 cases in the non-cough group showed that the factors related to CAP included surgical side (right lung), surgical lobe (upper lobe), preservation of pulmonary branch of the vagus nerve during operation, and length of anesthesia. These results are shown in **Table 5**.

**Table 5:** Univariate analysis of CAP.

General information		No CAP	CAP	x^2^/t/Fisher	P value
n		110	31		
Gender				1.162	0.281
	Male	59 (53.6)	20 (64.5)		
	Female	51 (46.4)	11 (35.5)		
Age (years)				0.554	0.457
	<60	58 (52.7)	14 (45.2)		
	≥60	52 (47.3)	17 (54.8)		
Length of anesthesia (min)		159.45 ± 33.05	180.65 ± 36.51	-3.081	0.002*
Operation time (min)		109.41 ± 33.73	122.74 ± 38.86	-1.879	0.062
Catheterization after operation (day)		3.77 ± 1.35	3.58 ± 0.92	0.743	0.459
Postoperative hospital stay		5.65 ± 1.68	5.42 ± 1.18	0.731	0.466
Intraoperative bleeding (ml)		87.27 ± 35.14	81.61 ± 32.47	0.805	0.422
The number of lymph nodes		6.23 ± 1.63	5.71 ± 1.35	1.619	0.108
Pathology				0.230	0.631
	Adenocarcinoma	108 (98.2)	30 (96.8)		
	Squamous cell carcinoma	2 (1.8)	1 (3.2)		
Pulmonary resection type				0.977	0.977
	Lobectomy	60 (54.5)	17 (54.8)		
	Subpulmonary lobectomy	50 (45.5)	14 (45.2)		
Operated side				5.445	0.020*
	Left lung	58 (52.7)	9 (29.0)		
	Right lung	52 (47.3)	22 (71.0)		
Tumor site				5.727	0.017*
	Upper lung	55 (50.0)	23 (74.2)		
	Middle and lower lung	55 (50.0)	8 (25.8)		
Past history					
	Hypertension	34 (30.9)	9 (29.0)	0.040	0.841
	Coronary heart disease	11 (10.0)	2 (6.5)	0.364	0.546
	Diabetes	15 (13.6)	3 (9.7)	0.078	0.560
	Smoking history	35 (31.8)	8 (25.8)	0.412	0.521
Treatment of pulmonary branch of vagus nerve				5.624	0.018*
	Pulmonary branch of vagus nerve preservation	62 (56.4)	10 (32.3)		
	Conventional surgical treatment	48 (43.6)	21 (67.7)		

^*^Statistically significant (p < 0.05).

### Multivariate Regression Analysis of CAP

Multivariate logistic regression analysis was performed on the statistically significant risk factors found in the aforementioned univariate analysis. The results indicated that there were significant correlations between length of anesthesia (OR: 1.024, 95% CI: 1.008-1.039, P=0.012), vagus nerve lung branch injury (OR: 2.525, 95% CI: 1.239-6.983, P =0.044), surgical side (OR: 2.747, 95% CI: 1.078-7.002, P = 0.034), and surgical side lobe (OR: 0.455, 95% CI: 0.178-0.978, P = 0.038) and CAP. That is, longer anesthesia time, vagus nerve lung branch injury, right lung surgery, and upper lobe surgery were each independent risk factors for CAP (P < 0.05). These results are shown in **Table 6**.

**Table 6:** Binary logistic regression analysis of CAP.

	b	SE	Wald	OR	95% CI	P
Length of anesthesia	0.023	0.008	8.697	1.024	1.008-1.039	0.012*
Vagus nerve lung branch injury	0.926	0.519	3.185	2.525	1.293-6.983	0.044*
Surgical side (right lung)	1.011	0.477	4.483	2.747	1.078-7.002	0.034*
Surgical side lobe (upper lobe)	-0.787	0.478	2.707	0.455	0.178-0.978	0.038*
Constant	-7.102	2.285	9.661	0.001		0.000*

OR, odds ratio; CI, confidence interval.

^*^Statistically significant (p < 0.05).

## Discussion

VATS is currently the best type of treatment for patients with early NSCLC (IA1-2) (17). The video-assisted thoracic surgery (VATS) has been widely used in early-stage non-small-cell lung cancer in recent years. We developed from open surgery to a minimally invasive surgery with three-, then two- and lastly uniportal VATS. Compared with anterolateral thoracotomy, VATS has significant advantages in postoperative pain and postoperative quality of life (18). However, CAP and other postoperative pulmonary complications are still the most common problems following treatment. The pulmonary vagus nerve plays important regulatory roles in both lung function and inflammation. Previous studies have shown that protection of the pulmonary vagus nerve branches during endoscopic surgery for esophageal cancer can effectively reduce postoperative pulmonary complications (19), but the relationship between the pulmonary vagus nerve and postoperative complications of VATS is not clear. At present, there is no previous report on protecting the pulmonary branch of the vagus nerve during VATS. To further verify the relationship between the pulmonary vagus nerve and postoperative complications of VATS, we prospectively protected the pulmonary vagus nerve branches during VATS.

In our experiment, we found no significant difference in complications between the two groups except for the occurrence of CAP. We used LCQ-MC and the VAS cough scale to evaluate postoperative CAP among our participants (20). The incidence of CAP in Group A was significantly lower than that of Group B (13.89% vs. 30.43%, P = 0.018). Our experimental results demonstrate that preserving the pulmonary vagus nerve branches during VATS can significantly reduce the incidence and severity of postoperative CAP. At the same time, this preservation did not reduce the incidence of other pulmonary complications.

There are many potential reasons that could explain these results. The pulmonary branch of the vagus nerve plays an important role in cough. The terminal of the vagus nerve innervates the distal airway and alveolar epithelial cells (21–23). It has a variety of mechanical and chemical receptors in this airway and can sense mechanical, chemical, and biological stimuli. The transient receptor potential channel vanillin subtype 1 (TRPV1) receptor is expressed on C fibers of the vagus nerve and is easily stimulated by chemical factors (24). Injury to the pulmonary branch of the vagus nerve during operation can promote the release of pneumonia factors (such as bradykinin and PGE2) on the affected side (25). After these inflammatory factors stimulate pulmonary mechanoreceptors, the TRPV1 pathway is activated and sends signals to the nerve center. This aggravates the persistent cough reflex, which is the basis of chronic cough (26). Preserving the pulmonary vagus nerve branches can effectively shorten this process.

According to the requirements of the 2018 NCCN guidelines for non-small cell lung cancer, systematic lymph node sampling is feasible for patients whose clinical stage is IA that they had been assessed completely before operation, it is possible to preserve the pulmonary vagus nerve branches during operation under the current technical level (27). Although it took a long time to preserve the pulmonary branch of the vagus nerve, the lymph node sampling time was shorter than that of the whole operation, so there was no significant difference in overall operation time. From our results, we conclude that it is safe and feasible to preserve the pulmonary branch of the vagus nerve during VATS.

Univariate and multivariate analysis of postoperative CAP patients showed that longer anesthesia time, intraoperative vagus nerve lung branch injury, right lung surgery, and upper lobe surgery were independent risk factors for CAP. The duration of anesthesia was the main influencing factor for chronic cough. A previous prospective clinical study has shown that laryngeal mask anesthesia in thyroid surgery had less stimulation on the throat than endotracheal intubation anesthesia, and could reduce the incidence of throat pain, cough, and hoarseness (28, 29). This finding suggests that endotracheal intubation can more easily damage tracheal mucosa and lead to airway inflammation. The longer the time of endotracheal intubation, the greater the stimulation of catheter balloon to the airway, resulting in edema and inflammation of tracheal tissue (30). The accumulation of acidic substances in the inflammatory reaction can directly stimulate the C fibers of the vagus nerve, excite the cough center, and induce bronchoconstriction and cough. In this experiment, double lumen endotracheal intubation under general anesthesia was used to eliminate the bias caused by using different anesthesia methods. Our findings confirmed that the longer the anesthesia was maintained, the more prone the patient was to chronic cough after operation, which was consistent with previous studies (31, 32).

Surgical factors are also one of the main causes of postoperative chronic cough. Our results suggested that right lung surgery and upper lobe surgery are more prone to postoperative CAP. The reason behind this may be CAP caused by bronchial torsion in the middle lobe of the right lung. Bronchial kinks are thought to be caused by the displacement of residual lobes. Previous studies have shown that the incidence of right middle bronchial kink after right upper lobectomy is higher than that after other lobectomy (33). This is consistent with the results of our experimental study.

In addition to pulmonary vagus nerve branches, we should pay attention to protecting recurrent laryngeal nerve during VATS. A recurrent laryngeal nerve injury during operation may lead to secondary unilateral vocal cord paralysis (UVFP). Previous studies have shown that Gore Tex implantation is an effective method for treating unilateral vocal cord paralysis after lung cancer surgery (34).Our study also has some limitations. Firstly, this is a prospective, single center study with a small sample size, meaning there may be bias in the results. Therefore, our findings need to be supported by multi-center randomized and double blinded controlled study research with larger sample sizes. Secondly, this experiment lacks long-term follow-up of postoperative CAP. Thirdly, the mechanism of preserving pulmonary vagus nerve branches to reduce the severity of CAP remains unclear and needs further research.

Robotic surgery has been gradually popularized in oral cancer surgery (35), chest surgery, and prostate cancer surgery. Such approach allows operators to magnify the operating field and respect the precise nervous structures. Therefore, robot assistance in thoracic surgery can maximize the benefits for patients. We intend to carry out further research in robotic surgery to verify the role of pulmonary vagus nerve branches in early lung cancer surgery (36).

In conclusion, preserving the pulmonary vagus nerve branches during VATS for early lung cancer can significantly reduce the incidence and severity of postoperative CAP, promote early postoperative rehabilitation, and improve the patient’s quality of life.

## Data Availability Statement

The raw data supporting the conclusions of this article will be made available by the authors, without undue reservation.

## Ethics Statement

The studies involving human participants were reviewed and approved by the ethics committee of Shanghai Tongji Hospital. The patients/participants provided their written informed consent to participate in this study.

## Author Contributions

The experimental design is completed by YZ and WW. WW and SX were the two surgeons who operated on the patients. SG, KW, and XW collected and analysed the data. XZ completed the randomization of patients. SG, SX, and YZ wrote the paper. All the authors contributed to the article and approved the submitted version.

## Funding

This project was supported by the Clinical Research Project of Tongji Hospital of Tongji University (Grant No. ITJ(ZD)1906),National Natural Science Foundation of China (no. 81974053),and Project of Shanghai Science and Technology Commission (No.20S31904700).

## Conflict of Interest

The authors declare that the research was conducted in the absence of any commercial or financial relationships that could be construed as a potential conflict of interest.

## Publisher’s Note

All claims expressed in this article are solely those of the authors and do not necessarily represent those of their affiliated organizations, or those of the publisher, the editors and the reviewers. Any product that may be evaluated in this article, or claim that may be made by its manufacturer, is not guaranteed or endorsed by the publisher.

## References

[1] Sihoe ADL. Video-Assisted Thoracoscopic Surgery as the Gold Standard for Lung Cancer Surgery. Respirology (2020) 25 Suppl 2:49–60. doi: 10.1111/resp.13920 32734596

[2] Harle ASM Blackhall FH Molassiotis A Yorke J Dockry R Holt KJ. Cough in Patients With Lung Cancer: A Longitudinal Observational Study of Characterization and Clinical Associations. Chest (2019) 155(1):103–13. doi: 10.1016/j.chest.2018.10.003 30321508

[3] Morice AH Jakes AD Faruqi S Birring SS McGarvey L Canning B. A Worldwide Survey of Chronic Cough: A Manifestation of Enhanced Somatosensory Response. Eur Respir J (2014) 44(5):1149–55. doi: 10.1183/09031936.00217813 25186267

[4] Li X Li X Zhang W Liu Q Gao Y Chang R. Factors and Potential Treatments of Cough After Pulmonary Resection: A Systematic Review. Asian J Surg (2021) 44(8):1029–36. doi: 10.1016/j.asjsur.2021.01.001 33610443

[5] Serra A Spinato G Spinato R Conti A Licciardello L Di Luca M. Multicenter Prospective Crossover Study on New Prosthetic Opportunities in Post-Laryngectomy Voice Rehabilitation. J Biol Regul Homeost Agents (2017) 31(3):803–9. 28958139

[6] Li S Qi D Li JN Deng XY Wang DX. Vagus Nerve Stimulation Enhances the Cholinergic Anti-Inflammatory Pathway to Reduce Lung Injury in Acute Respiratory Distress Syndrome *via* STAT3. Cell Death Discov (2021) 7(1):63. doi: 10.1038/s41420-021-00431-1 33782389 PMC8005666

[7] Andersson U. The Cholinergic Anti-Inflammatory Pathway Alleviates Acute Lung Injury. Mol Med (2020) 26(1):64. doi: 10.1186/s10020-020-00184-0 32600316 PMC7322708

[8] Cheng X Chen H. Commentary on the Impacts of Postoperative Complications on Survival After Lung Cancer Surgery. J Thorac Cardiovasc Surg (2018) 155(3):1265–6. doi: 10.1016/j.jtcvs.2017.10.122 29198796

[9] Weijs TJ Goense L van Rossum PS Meijer GJ van Lier AL Wessels FJ. Theperi-Esophageal Connective Tissue Layers and Related Compartments: Visualization by Histology and Magnetic Resonance Imaging. J Anat (2017) 230:262–71. doi: 10.1111/joa.12552 PMC5244460 27659172

[10] Weijs TJ Berkelmans GH Nieuwenhuijzen GA Dolmans AC Kouwenhoven EA Rosman C. Immediate Postoperative Oral Nutrition Following Esophagectomy: A Multicenter Clinical Trial. Ann Thorac Surg (2016) 102:1141–8. doi: 10.1016/j.athoracsur.2016.04.067 27324526

[11] Chang SH Kang YN Chiu HY Chiu YH. A Systematic Review and Meta-Analysis Comparing Pigtail Catheter and Chest Tube as the Initial Treatment for Pneumothorax. Chest (2018) 153(5):1201–12. doi: 10.1016/j.chest.2018.01.048 29452099

[12] Bendixen M Jorgensen OD Kronborg C Andersen C Licht PB. Postoperative Pain and Quality of Life After Lobectomy *via* Video-Assisted Thoracoscopic Surgery or Anterolateral Thoracotomy for Early Stage Lung Cancer: A Randomised Controlled Trial. Lancet Oncol (2016) 17(6):836–44. doi: 10.1016/S1470-2045(16)00173-X 27160473

[13] Ettinger DS Aisner DL Wood DE Akerley W Bauman J Chang JY. Nccn Guidelines Insights: Non-Small Cell Lung Cancer, Version 5.2018. J Natl Compr Canc Netw (2018) 16(7):807–21. doi: 10.6004/jnccn.2018.0062 30006423

[14] Chansky K Detterbeck FC Nicholson AG Rusch VW Vallieres E Groome P. The IASLC Lung Cancer Staging Project: External Validation of the Revision of the TNM Stage Groupings in the Eighth Edition of the TNM Classification of Lung Cancer. J Thorac Oncol (2017) 12(7):1109–21. doi: 10.1016/j.jtho.2017.04.011 28461257

[15] Lin R Che G. Validation of the Mandarin Chinese Version of the Leicester Cough Questionnaire in Non-Small Cell Lung Cancer Patients After Surgery. Thorac Cancer (2018) 9(4):486–90. doi: 10.1111/1759-7714.12602 PMC5879047 29484851

[16] Sung YT Wu JS. The Visual Analogue Scale for Rating, Ranking and Paired-Comparison (VAS-RRP): A New Technique for Psychological Measurement. Behav Res Methods (2018) 50(4):1694–715. doi: 10.3758/s13428-018-1041-8 PMC6096654 29667082

[17] Klapper J D’Amico TA. VATS Versus Open Surgery for Lung Cancer Resection: Moving Toward a Minimally Invasive Approach. J Natl Compr Canc Netw (2015) 13(2):162–4. doi: 10.6004/jnccn.2015.0023 25691607

[18] Migliore M Calvo D Criscione A Borrata F. Uniportal Video Assisted Thoracic Surgery: Summary of Experience, Mini-Review and Perspectives. J Thorac Dis (2015) 7(9):E378–80. doi: 10.3978/j.issn.2072-1439.2015.07.35 PMC4598499 26543631

[19] Weijs TJ Ruurda JP Luyer MD Nieuwenhuijzen GA van Hillegersberg R Bleys RL. Topography and Extent of Pulmonary Vagus Nerve Supply With Respect to Transthoracic Oesophagectomy. J Anat (2015) 227:431–9. doi: 10.1111/joa.12366 PMC4580102 26352410

[20] Lin R Che G. Risk Factors of Cough in Non-Small Cell Lung Cancer Patients After Video-Assisted Thoracoscopic Surgery. J Thorac Dis (2018) 10(9):5368–75. doi: 10.21037/jtd.2018.08.54 PMC6196164 30416784

[21] Chen C Zhang Y Du Z Zhang M Niu L Wang Y. Vagal Efferent Fiber Stimulation Ameliorates Pulmonary Microvascular Endothelial Cell Injury by Downregulating Inflammatory Responses. Inflammation (2013) 36(6):1567–75. doi: 10.1007/s10753-013-9701-4 23912647

[22] Umans BD Liberles SD. Neural Sensing of Organ Volume. Trends Neurosci (2018) 41(12):911–24. doi: 10.1016/j.tins.2018.07.008 PMC6252275 30143276

[23] Belvisi MG. Overview of the Innervation of the Lung. Curr Opin Pharmacol (2002) 2(3):211–5. doi: 10.1016/S1471-4892(02)00145-5 12020459

[24] Mazzone SB McGarvey L. Mechanisms and Rationale for Targeted Therapies in Refractory and Unexplained Chronic Cough. Clin Pharmacol Ther (2021) 109(3):619–36. doi: 10.1002/cpt.2003 PMC7983941 32748976

[25] Ho CY Gu Q Hong JL Lee LY. Prostaglandin E(2) Enhances Chemical and Mechanical Sensitivities of Pulmonary C Fibers in the Rat. Am J Respir Crit Care Med (2000) 162(2 Pt 1):528–33. doi: 10.1164/ajrccm.162.2.9910059 10934082

[26] Al-Shamlan F El-Hashim AZ. Bradykinin Sensitizes the Cough Reflex *via* a B2 Receptor Dependent Activation of TRPV1 and TRPA1 Channels Through Metabolites of Cyclooxygenase and 12-Lipoxygenase. Respir Res (2019) 20(1):110. doi: 10.1186/s12931-019-1060-8 31170972 PMC6551914

[27] Zhong WZ Liu SY Wu YL. Numbers or Stations: From Systematic Sampling to Individualized Lymph Node Dissection in Non-Small-Cell Lung Cancer. J Clin Oncol (2017) 35(11):1143–5. doi: 10.1200/JCO.2016.71.8544 28380312

[28] Minogue SC Ralph J Lampa MJ. Laryngotracheal Topicalization With Lidocaine Before Intubation Decreases the Incidence of Coughing on Emergence From General Anesthesia. Anesth Analg (2004) 99(4):1253–7. doi: 10.1213/01.ANE.0000132779.27085.52 15385385

[29] Wu CY Chen JS Lin YS Tsai TM Hung MH Chan KC. Feasibility and Safety of Nonintubated Thoracoscopic Lobectomy for Geriatric Lung Cancer Patients. Ann Thorac Surg (2013) 95(2):405–11. doi: 10.1016/j.athoracsur.2012.10.082 23245449

[30] Gibson PG Vertigan AE. Management of Chronic Refractory Cough. BMJ (2015) 351:h5590. doi: 10.1136/bmj.h5590 26666537

[31] White PF Tang J Wender RH Yumul R Stokes OJ Sloninsky A. Desflurane Versus Sevoflurane for Maintenance of Outpatient Anesthesia: The Effect on Early Versus Late Recovery and Perioperative Coughing. Anesth Analg (2009) 109(2):387–93. doi: 10.1213/ane.0b013e3181adc21a 19608808

[32] Pan LY Peng LP Xu C Ding C Chen J Wang WY. Predictive Factors of Cough After Uniportal Video-Assisted Thoracoscopic Pulmonary Resection. J Thorac Dis (2020) 12(10):5958–69. doi: 10.21037/jtd-20-2652 PMC7656438 33209428

[33] Gu Q Qi S Yue Y Shen J Zhang B Sun W. Structural and Functional Alterations of the Tracheobronchial Tree After Left Upper Pulmonary Lobectomy for Lung Cancer. BioMed Eng Online (2019) 18(1):105. doi: 10.1186/s12938-019-0722-6 31653252 PMC6815003

[34] Cocuzza S Di Luca M Maniaci A Russo M Di Mauro P Migliore M. Precision Treatment of Post Pneumonectomy Unilateral Laryngeal Paralysis Due to Cancer. Future Oncol (2020) 16(16s):45–53. doi: 10.2217/fon-2019-0053 31912750

[35] Meccariello G Maniaci A Bianchi G Cammaroto G Iannella G Catalano A. Neck Dissection and Trans Oral Robotic Surgery for Oropharyngeal Squamous Cell Carcinoma. Auris Nasus Larynx (2021) 49(1):17–25. doi: 10.1016/j.anl.2021.05.007 34092436

[36] Schwartz G Sancheti M Blasberg J. Robotic Thoracic Surgery. Surg Clin North Am (2020) 100(2):237–48. doi: 10.1016/j.suc.2019.12.001 32169178

